# Evaluation of Fluid Responsiveness: Is Photoplethysmography a Noninvasive Alternative?

**DOI:** 10.1155/2012/617380

**Published:** 2012-02-28

**Authors:** Lars Prag Antonsen, Knut Arvid Kirkebøen

**Affiliations:** ^1^Faculty of Medicine, University of Oslo, 0316 Oslo, Norway; ^2^Department of Anesthesiology, Oslo University Hospital, Ullevaal, 0424 Oslo, Norway

## Abstract

*Background*. Goal-directed fluid therapy reduces morbidity and mortality in various clinical settings. Respiratory variations in photoplethysmography are proposed as a noninvasive alternative to predict fluid responsiveness during mechanical ventilation. This paper aims to critically evaluate current data on the ability of photoplethysmography to predict fluid responsiveness. *Method*. Primary searches were performed in PubMed, Medline, and Embase on November 10, 2011. *Results*. 14 papers evaluating photoplethysmography and fluid responsiveness were found. Nine studies calculated areas under the receiver operating characteristic curves for ΔPOP (>0.85 in four, 0.75–0.85 in one, and <0.75 in four studies) and seven for PVI (values ranging from 0.54 to 0.98). Correlations between ΔPOP/PVI and ΔPP/other dynamic variables vary substantially. *Conclusion*. Although photoplethysmography is a promising technique, predictive values and correlations with other hemodynamic variables indicating fluid responsiveness vary substantially. Presently, it is not documented that photoplethysmography is adequately valid and reliable to be included in clinical practice for evaluation of fluid responsiveness.

## 1. Introduction

Whether or not to administer intravenous (iv) fluid is a common, difficult, and controversial challenge in clinical practice. The main aim of fluid therapy during surgery or critical illness is to provide adequate tissue perfusion by increasing stroke volume (SV) or cardiac output (CO). Goal-directed fluid therapy aiming to increase oxygen (O_2_) delivery reduces morbidity and mortality in various clinical settings [1–8]. Fluid therapy is guided by clinical variables, as well as static and dynamic variables. Clinical variables include blood pressure, heart rate, capillary refill time, skin turgor and diuresis, mixed venous oxygen saturation (SvO_2_), lactate, pH, electrolytes, and creatinine/urea. Conventional static variables include central venous pressure (CVP) and pulmonary artery wedge pressure (PAWP), but these variables have proven less reliable than initially assumed to evaluate fluid responsiveness [8–10]. Dynamic variables include both SV-dependent and non-SV-dependent methods. The ideal new method should be accurate [11], easy to use, noninvasive, and widely available with minimal risk of complications. Potential clinical value also depends on reproducibility and predictive values compared to established methods.

Photoplethysmography (more specifically pulse oximetry plethysmographic waveform analysis) as a noninvasive tool in evaluation of fluid responsiveness was first described by Partridge [12] and has been extensively investigated. A pulse oximeter is a standard equipment for measuring arterial O_2_ saturation, and further analysis of the photoplethysmographic signal can easily be implemented in clinical monitoring. This paper aims to critically evaluate current data on the ability of photoplethysmography to predict fluid responsiveness.

## 2. Methods

This paper is based on searches performed in PubMed, Medline, and Embase on November 10, 2011 with the following search criteria: “(pulse oximetry OR plethysmographic OR Pleth variability index OR PVI) AND ((fluid responsiveness) OR (volume status)).” The searches generated 217 hits. Papers were checked for relevant references and 22 [13–34] papers met the following inclusion criteria:

1. reporting predictive values of ΔPOP and/or PVI after fluid challenges and/or reporting correlations between ΔPOP, PVI, and ΔPP,
2. mechanically ventilated patients,
3. written in English.

## 3. Results

### 3.1. Predictive Values of ΔPOP and PVI

14 studies performed fluid challenges and these are summarized in Table 1. Patients were mechanically ventilated with tidal volumes of 6–10 mL/kg and investigated preoperatively (*n* = 6) [23, 26, 28, 30–32], perioperatively (*n* = 3) [21, 33, 34], postoperatively (*n* = 2) [25, 27], and in the intensive care unit (ICU) (*n* = 2) [22, 24]. One study included different groups of patients [29]. The pulse oximeter was placed on a finger in all papers, and in two papers it was also placed on the earlobe [30, 34]. Different types of pulse oximeters were used, summarized in Table 2. The number of patients included in each study varied substantially (*n* = 8–43). Registration periods were, with some exceptions [22, 33], short (<1 min). Six studies did not indicate duration of the registration period [24, 28–31, 34]. Patients with arrhythmias were excluded in all papers except one, in which this was not expliticitly stated [34]. Patients receiving vasoactive medication were included in four papers [24, 27, 29, 33] and excluded in four [23, 26, 31, 32]. Six papers did not indicate use of vasoactive medication [21, 22, 25, 28, 30, 34]. These data are summarized in Table 3. Fluid challenges were given as HES 6% (*n* = 9) [22–26, 28–31], “colloid fluid” (*n* = 3) [21, 33, 34], and NaCl 0.9% (*n* = 2) [27, 32]. Fluid volumes ranged from 250 mL to 1000 mL. Fluid responsiveness was defined as increased CO > 15% (*n* = 2) [25, 29], increased CI > 15% (*n* = 5) [22–24, 26, 30], ΔPP > 13% (*n* = 1) [27], increased SVI > 10% (*n* = 1) [21], increased SVI > 15% (*n* = 2) [28, 31], increased SV > 10% (*n* = 1) [34], increased SV > 15% (*n* = 1) [33], and aortic velocity-time integral (AVTI) > 15% (*n* = 1) [32]. Best cut-off values ranged from 8.8 to 15% for ΔPP, 9.5 to 15%  for ΔPOP, and 9.5 to 17% for PVI. CO was measured with thermodilution (*n* = 5) [21–23, 26, 30], echo Doppler (*n* = 6) [24, 29, 31–34], FloTrac/Vigileo (*n* = 1) [28], and intermittent thermodilution by pulmonary artery catheter (Vigilance monitor) (*n* = 1) [25].

Nine studies calculated areas under receiver operating characteristics curves (ROC curves) for ΔPOP [21–27, 32, 33]. It was calculated to >0.85 in four studies [24–27], 0.75–0.85 in one [23], and <0.75 in four [21, 22, 32, 33]. In five studies values for ΔPOP were as good as, or better than, values for ΔPP [23, 24, 26, 27, 33]. In one of these studies, predictive value of ΔPOP was defined as a certain change in ΔPP, thus presuming that ΔPP is a good indicator [27]. One study found poor values for both ΔPP and ΔPOP [33]. Four studies reported lower predictive values for ΔPOP than for ΔPP [21, 22, 25, 32]. Thus, only two of nine studies reported high predictive values for ΔPOP [24, 26]. The ROC curves for ΔPOP and ΔPP were not found to be significantly different in any of the nine studies. The best ΔPOP cut-off value for identifying responders ranged from 9.5 to 15%.

Seven studies calculated ROC curves for PVI [26, 28–32, 34], with values ranging from 0.54 to 0.98. Although correlations between PVI and other parameters vary, predictive values remain relatively good in stable conditions. In one study, the predictive value of PVI decreased from 0.96 at baseline to 0.71 perioperatively [34]. The best PVI cut-off value for identifying responders ranged from 9.5 to 17%.

### 3.2. Correlations between ΔPOP, PVI, and ΔPP

ΔPP is considered to be a good predictor of fluid responsiveness [6]. Thus, other variables should correlate with ΔPP. 11 of the included papers reported correlations between ΔPP and ΔPOP. Six of these papers reported relatively good correlations (*r* > 0.84) [13, 15, 17, 23, 24, 27]. However, five papers reported relatively poor correlations (*r* < 0.78) [14, 16, 18, 32, 33]. One of these investigated children preoperatively [32]. Landsverk et al. [16] concluded that there are poor correlations between ΔPOP and ΔPP in ICU patients due to sympathetic oscillations in skin circulation, which lead to larger variation in ΔPOP than in ΔPP during registrations over longer time periods. These findings are supported by Hoiseth et al. [33] who also found larger variation in ΔPOP than in ΔPP during ongoing open major abdominal surgery. Four papers examined correlations between PVI and ΔPP. Three of them found relatively poor correlations (*r* = 0.72, 0.46 and 0.78) [17, 20, 32], whereas one reported better correlations (*r* = 0.85) [29]. Three papers investigated correlations between PVI and ΔPOP [17, 26, 32]. One study reported poor correlations (*r* = 0.39) [32], whereas two studies reported relatively good correlations (*r* = 0.92) [17, 26]. These data are presented in Table 2.

## 4. Discussion

Photoplethysmography is applicable on most patient categories and is noninvasive, simple, widely available, and without risk of complications. Several physiological, clinical, and practical factors must be taken into account when evaluating whether or not it is a noninvasive alternative to evaluate fluid responsiveness.

Firstly, there are several physiological prerequisitions for using dynamic variables.

Mechanical ventilation provides the stable and predictable variations in intrathoracic pressure required for photoplethysmography to be accurate. A large mechanical tidal volume will influence intrathoracic pressure to a greater extent than a small tidal volume. It is presumed that the influence of tidal volume reaches significance at >8 mL/kg. It is a challenge that the accuracy of photoplethysmography increases with larger tidal volumes, whereas it is clinically desirable to minimize the tidal volume. The accuracy of photoplethysmography relies on a continuous beat-to-beat-analysis. Thus, patients need to have stable heart rate. Additionally, decreased RV ejection fraction can lead to false-positive variations in pulse pressure [35]. These requisitions also apply for other dynamic variables [36–39].

Secondly, the complex network of correlations between ΔPOP/PVI and ΔPP/other hemodynamic variables varies greatly between different studies. The best correlations are found in studies where short registration periods (3–5 respiratory cycles) have been used and in patients under stable pre- and postoperative conditions. These conditions do not reflect genuine intraoperative instability, the setting where precise guidance of fluid therapy is perhaps most important. The correlations are poorer with longer periods of registration [16], in heterogeneous patient groups in ICUs [16], and during ongoing open abdominal surgery [21, 33]. The best predictive values for ΔPOP and PVI were found in papers in which patients were investigated preoperatively [26, 28]. The poorest predictive values (0.51–0.72) were found during ongoing open major abdominal surgery [21, 33], on sedated patients in ICU [22], and on children preoperatively [32]. In one paper, the predictive value of PVI decreased from 0.96 at baseline to 0.71 during surgery [34]. This indicates that photoplethysmography shows best results in standardized conditions, during short registration periods, and in homogenous groups of pre- and postoperative patients. Importantly, it has been demonstrated that PVI reduces both lactate levels and volumes of fluid administered in surgical patients [40]. This is interesting evidence. However, the study does not report improvement in terms of the number of complications. Further studies are needed to clarify the very important aspect of improved outcome.

Finally, a number of additional factors must be considered. Variations in total peripheral resistance and vasomotor tone increase under the influence of general anesthesia [41, 42], with vasoactive drugs, with site of measurement, and with physiological responses such as inflammation, pain, fear, and body temperature. This may lead to inaccuracy of the photoplethysmography signal. The papers included suggest that ΔPOP is less reliable in ICU patients. This may be explained by the above-mentioned factors. Hemodynamics of patients in the OR or in ICUs changes rapidly and continuously. In most papers which good predictive values for photoplethysmography have been found, short registration periods are used. In papers with longer registration periods, poorer predictive values have been reported.

A threshold value refers to a value of ΔPOP, ΔPP, or PVI that separates responders from nonresponders. Failure to agree upon a threshold value in clinical settings does not necessarily make the parameters (i.e., PVI or POP) less valuable. Different patient groups may well present with different threshold values. A septic patient may have a threshold value different from that of a hemodynamically stable patient undergoing surgery. In the same way, threshold values may also change pre-, peri-, and postoperatively. Cannesson et al. [43] discussed the very interesting notion of a gray-zone approach to fluid responsiveness and found that an intermediate zone of pulse pressure variation could not predict fluid responsiveness. Future studies should grade responses instead of dividing responses in two categories.

Cut-off values for increases in SV/CO/CI are defined to separate reponders and nonresponders. These thresholds are based on the variability and errors in the chosen measuring technique as well as what change is believed to be clinically important. These thresholds may be more or less arbitrarily chosen and differ between the studies.

 Level of intra-abdominal pressure may influence ΔPP and ΔPOP and is relevant in three of the articles included [21, 28, 33]. Results are not coherent. Animal studies have shown that increased intra-abdominal pressure leads to an increase in ΔPP [44]. Studies investigating the influence of these fluctuations during laparoscopic surgery are currently running.

In theory, a number of potentially confounding factors exist. Different pulse oximeter-technology, errors due to software autogain features which filter and amplify the raw signal (thus making it unreliable for quantitative analysis), atherosclerosis, type of fluid, skin pigmentation, saturation, movement artefacts, statistical weaknesses, variations in pleural and transpulmonary pressures, and venous components of the pulsatile signal may affect measurements.

## 5. Conclusion

We conclude that although photoplethysmography is a promising technique, predictive values and correlations with other hemodynamic variables indicating fluid responsiveness vary substantially. Based on studies using short registration periods photoplethysmography might seem promising for evaluation of volume status. However, in studies using longer registration periods it has been shown that intra- and interindividual variability for ΔPOP is greater than for ΔPP, leading to poor agreement between ΔPOP and ΔPP. Thus, it is not presently evident that photoplethysmography is adequately accurate, valid, and reliable to be included in clinical practice for evaluation of volume status. In future studies it is important to evaluate new hemodynamic methods in clinically relevant settings and to test their reproducibility in clinically relevant time frames. Relatively poor predictive values during ongoing major surgery further underscore this point and results vary in different patient groups. The greatest potential for photoplethysmography in evaluation of volume status might be in settings where invasive monitoring is not indicated.

## Figures and Tables

**Table 1:** Papers in which ΔPOP and/or PVI have been evaluated and fluid challenges performed.

Author, Ref.	Fluid challenge	CO/CI/SVI measurement	Responder	ROC	Threshold value	Sens/spec
Solus-Biguenet et al. [21]	250 mL colloid	Thermodilution	SVI: 10%	0.81	PPV_fina_: 14%	No data
				0.68	ΔPOP: 9.5%	No data
				0.79	PPV_art_: 12.5%	No data
Natalini et al. [22]	500 mL HES 6%	Thermodilution	CI: 15%	0.72	ΔPOP: 15%	56/86 (PPV/NPV)
				0.74	ΔPP: 15%	55/100 (PPV/NPV)
Cannesson et al. [23]	500 mL HES 6%	Thermodilution	CI: 15%	0.847	ΔPOP: 13%	93/90
				0.847	ΔPP: 11%	80/90
Feissel et al. [24]	8 mL/kg HES 6%	Echo-Doppler	CI: 15%	0.94	ΔPP: 12%	100/70
				0.94	ΔPOP: 14%	94/80
Wyffels et al. [25]	500 mL HES 6%	Intermittent thermodilution	CO: 15%	0.94	PPV: 11.3%	95/91.7
		by pulm. artery catheter		0.89	ΔPOP: 11.3%	90/83.3
Cannesson et al. [26]	500 mL HES 6%	Thermodilution	CI: 15%	0.94	ΔPP: 12.5%	87/89
				0.94	ΔPOP: 12%	87/89
				0.93	PVI: 14%	81/100
Westphal et al. [27]	500–1000 mL NaCl	Not measured	ΔPP > 13%	0.95	ΔPOP: 11%	91/100
Zimmermann et al. [28]	7 mL/kg HES 6%	FloTrac	SVI: 15%	0.97	PVI: 9.5 %	93/100
Loupec et al. [29]	500 mL HES	Echocardiography	CO: 15%	0.88	PVI: 17%	95/91
	or PLR if ΔPP < 13%					
Desgranges et al. [30]	500 mL HES	Thermodilution	CI: 15%	0.91	PVI_forehead _: 15%	89/78
				0.88	PVI_ear_: 16%	74/74
				0.84	PVI_finger _: 12%	74/67
				0.84	PPV > 11%	74/89
					PVI_forehead _: 15%	89/100
					and PI_forehead _: 1.37	
Renner et al. [31]	HES 10 mL kg^−1^	Transoesophageal echocardiography	SVI: 15%	0.79	PVI > 13%	84/64
De Souza Neto et al. [32]	Saline, 20 mL/kg	Transthoracic echography	AVTI: 15%	0.51	0–6 yr: ΔPOP	No data
		(aortic velocity-time integral)		0.63	0–6 yr: PVI	No data
				0.71	0–6 yr: ΔPP	No data
				0.52	0–6 yr: PPV	No data
				0.57	6–14 yr: ΔPOP	No data
				0.54	6–14 yr: PVI	No data
				0.60	6–14 yr: ΔPP	No data
				0.60	6–14 yr: PPV	No data
Hoiseth et al. [33]	250 mL colloid	Esophageal doppler	SV ≥ 15%	0.67	ΔPP: 8.8%	82/67
				0.72	ΔPOP: 11.4%	86/67
Hood and wilson [34]	500 mL colloid	Esophageal doppler	SV ≥ 10%	0.96	PVI_finger _ (baseline) 10%	86/100
				0.98	PVI_earlobe _ (baseline) 9.5%	95/100
				0.71	PVI_finger _ (during surgery) 10%	65/67
				0.54	PVI_earlobe _ (during surgery)	

ΔPOP: pulse oximetry plethysmography; ΔPP: pulse pressure; PVI: Pleth variability index; CI: cardiac index; SV: stroke volume; SVI: stroke volume index; SVV: stroke volume variation; CO: cardiac output; PPVfina: pulse pressure variation obtained with Finapres; PPVart: pulse pressure variation obtained with intraarterial equipment; PPV/NPV: positive predictive value/negative predictive value.

**Table 2:** Papers in which correlations between ΔPOP, PVI, and ΔPP have been investigated.

Author, Ref.	Relation	Correlation	Pulse oximeter/monitor
Cannesson et al. [13]	ΔPOP-ΔPP	*r* = 0.91, *P* < 0.001	M1190A, Philips, Suresnes, France
Natalini et al. [14]	ΔPOP-ΔPP	*r* = 0.62, *P* < 0.001	Datex-Engstrom CS/3 Critical Care Monitor, Instrumentarium, Helsinki, Finland
Cannesson et al. [15]	ΔPOP-ΔPP	*r* = 0.89, *P* < 0.01	Oxymax Tyco Healthcare Group LP, Pleasanton, CA, USA
			Intellivue MP70, Philips Medical Systems, Suresnes, France
Landsverk et al. [16]	ΔPOP-ΔPP	*r* = 0.05, *P* = 0.15	OxiMax 451N5, Nellcor, Boulder, CO, USA
Cannesson et al. [17]	ΔPOP-PVI	*r* = 0.92, *P* < 0.05	LNOP Adt, Masimo Corp., Irvine, CA, USA
	PVI-ΔPP	*r* = 0.72, *P* < 0.05	Oxymax, Tyco Healthcare Group LP, Pleasanton, CA, USA
	ΔPOP-ΔPP	*r* = 0.86, *P* < 0.05	Intellivue MP70, Philips Medical Systems, Suresnes, France
Pizov et al. [18]	ΔPOP-ΔPP	*r* = 0.75	Datex-Ohmeda AS-3, Datex, Helsinki, Finland
Desebbe et al. [19]	PVI_ VT = 6_-PVI_ VT = 10_	9%–12%, *P* = 0.001	LNOP Adt, Masimo Corp., Irvine, CA, USA
	PVI_PEEP_- PVI_non - PEEP _	(Significant change)	
Biais et al. [20]	PVI-ΔPP	*r* = 0.46, *P* = 0.001	LNOP Adt, Masimo Corp., Irvine, CA, USA
	PVI_NE(+)_-ΔPP	*r* = 0.20, *P* > 0.05	Masimo Radical 7 monitor, Masimo SET, Masimo Corp., Irvine, CA, USA
	PVI_NE(−)_-ΔPP	*r* = 0.72, *P* < 0.001	
Solus-Biguenet et al. [21]			No data
Natalini et al. [22]			Datex-Engstrom CS/3 Critical Care Monitor, Instrumentarium, Helsinki, Finland
Cannesson et al. [23]	ΔPOP-ΔPP	*r* = 0.90, *P* < 0.01,	Oxymax Tyco Healthcare Group LP, Pleasanton, CA, USA
			Intellivue MP70, Philips Medical Systems, Suresnes, France
Feissel et al. [24]	ΔPOP-ΔPP	*r* = 0.84, *P* < 0.001	SpO2/Pleth, M3150A technology, Philips Medical Systems, Andover, MA, USA
Wyffels et al. [25]			Monitor Hewlett Packard M1166A model G65
Cannesson et al. [26]	ΔPOP-PVI	*r* = 0.92, *P* < 0.01	LNOP Adt, Masimo Corp., Irvine, CA, USA, with Masimo Radical 7, 7.0.3.3
			Oxymax, Tyco Healthcare Group LP, Pleasanton, CA, USA
Westphal et al. [27]	ΔPOP-ΔPP	*r* = 0.90, *P* < 0.001	S/5, Datex-Ohmeda, Helsinki, Finland
Zimmermann et al. [28]			LNCS, Masimo Corp., Irvine, CA, USA, Masimo Radical-7 monitor, 7.0.3.3
Loupec et al. [29]	PVI_baseline _-ΔPP_baseline _	*r* = 0.85, *P* < 0.0001	LNCS Adtx, Masimo corp., Irvine, CA, USA
Desgranges et al. [30]			LNOP Adt, Masimo Corp., Irvine, CA, USA
			LNOP TC-I, Masimo Corp., Irvine, CA, USA
			LNOP TF-I, Masimo Corp., Irvine, CA, USA
			Masimo Radical 7, Masimo SET, Masimo Corp., version 7.1.1.5
Renner et al. [31]			Masimo Rainbow SET, Masimo Corp., Radical 7, V7.6.2.2
De Souza et al. [32]	ΔPOP-PVI	*r* = 0.39, *P* < 0.05	Oxymax*™*, Tyco Healthcare Group LP, Pleasanton, CA, USA
	ΔPOP-ΔPP	*r* = 0.48, *P* < 0.001	LNOP, Masimo Corp., Irvine, CA, USA
	PVI-PPV	*r* = 0.78, *P* < 0.001	
Hoiseth et al. [33]	ΔPOP-ΔPP	*r* = 0.78, *P* < 0.001	OxiMax 451N5, Nellcor, Boulder, CO, USA
Hood and wilson [34]			Masimo Rainbow SET, Masimo Corp., Irvine, CA, USA

ΔPOP: pulse oximetry plethysmography; ΔPP: pulse pressure; PVI: Pleth variability index; PEEP: positive end expiratory pressure; NE: norepinephrine; VT: tidal volume.

**Table 3:** General characteristics.

Author, Ref.	Year	*n*	Patient category	Ventilation	Site of meas.	Reg. period	Vasoact.
Cannesson et al. [13]	2005	22	ICU	Mech. vent. 6–10 mL/kg, volume	Finger	3 respiratory cycl.	Incl.
Natalini et al. [14]	2006	49	OR/ICU	Mech. vent. 6–9 mL/kg, volume	Finger/toe	5 respiratory cycl.	No data
Cannesson et al. [15]	2007	25	Preop. CABG/AAA	Mech. vent. 8–10 mL/kg, volume	Finger	3 respiratory cycl.	Excl.
			Gen.anaesthesia				
Landsverk et al. [16]	2008	14	ICU	Mech. vent. 8 mL/kg, volume/pressure	Finger	15 min	Incl.
Cannesson et al. [17]	2008	25	Preop.CABG	Mech. vent. 8–10 mL/kg	Finger	3 respiratory cycl.	Excl.
			Gen.anaesthesia				
Pizov et al. [18]	2010	33	Preop. surgery	Mech. vent. 8–10 mL/kg	Finger	3 min	Incl.
Desebbe et al. [19]	2010	21	Postop. CABG and ICU	Mech. vent. 6–10 mL/kg, volume	Finger	3 respiratory cycl.	Excl.
Biais et al. [20]	2011	67	ICU	Mech. vent. 8 mL/kg, volume	Finger	3 respiratory cycl.	Incl.

Solus-Biguenet et al. [21]	2006	8	During hepatic surgery	Mech. vent. 8–10 mL/kg	Finger	3 respiratory cycl.	No data
Natalini et al. [22]	2006	22	ICU	Mech. vent. 6–10 mL/kg, volume	Finger	2 min	No data
Cannesson et al. [23]	2007	25	Preop. CABG	Mech. vent. 8–10 mL/kg, volume	Finger	3 respiratory cycl.	Excl.
Feissel et al. [24]	2007	23	ICU	Mech. vent. 8 mL/kg, pressure	Finger	No data	Incl.
Wyffels et al. [25]	2007	32	Postop. heart surgery	Mech. vent. 8–10 mL/kg	Finger	3 respiratory cycl.	No data
Cannesson et al. [26]	2008	25	Preop.CABG	Mech. vent. 8–10 mL/kg, volume	Finger	3 respiratory cycl.	Excl.
			Gen.anaesthesia				
Westphal et al. [27]	2009	43	Postop. heart surgery	Mech. vent. 8–10 mL/kg, volume	Finger	1 min	Incl.
Zimmermann et al. [28]	2010	20	Preop. abd. surgery	Mech. vent. 7 mL/kg, volume	Finger	No data	No data
Loupec et al. [29]	2011	40	Several categories	Mech. vent. 8 mL/kg, volume	Finger	No data	Incl.
Desgranges et al. [30]	2011	28	Preop. cardiac surgery	Mech. vent. 8 mL/kg, volume	Finger, earlobe	No data	No data
					and forehead		
Renner et al. [31]	2011	27	Infants preop. cardiac surgery	Mech. vent. 10 mL/kg, volume	No data	No data	Excl.
Pereira de Souza et al. [32]	2011	30	Children preop neurosurgery	Mech. vent. 10 mL/kg, volume	Finger	3 respiratory cycl.	Excl.
Hoiseth et al. [33]	2011	25	During abd. surgery	Mech. vent. 8 mL/kg, volume	Finger	App. 5 min	Incl.
Hood and wilson [34]	2011	25	During colorectal surgery	Mech. vent. 8–10 mL/kg, volume	Finger/earlobe	No data	No data

ICU: intensive care unit; OR: operating room; CABG: coronary artery bypass grafting; AAA: abdominal aortic aneurysm*.*
